# Following in Banting’s footsteps or straying from the path? Observations from contemporary diabetes innovation

**DOI:** 10.3389/fendo.2023.1270517

**Published:** 2023-11-14

**Authors:** Connor Leadley, Ananta Addala, Juliet Berkeley, Hamish Crocket, Elizabeth A. Davis, Niranjala Hewapathirana, Sufyan Hussain, Rayhan Lal, Kate Lomax, Thomas Wilkinson, Martin de Bock, Marie-Anne Burckhardt

**Affiliations:** ^1^ Department of Pediatrics, University of Otago, Christchurch, New Zealand; ^2^ Division of Endocrinology, Department of Pediatrics, Stanford University, Stanford, United States; ^3^ Stanford Diabetes Research Center, Stanford University, Stanford, United States; ^4^ Department of Diabetes and Endocrinology, Te Whatu Ora Waitaha, Christchurch, New Zealand; ^5^ Te Huataki Waiora School of Health, University of Waikato, Hamilton, New Zealand; ^6^ Department of Endocrinology and Diabetes, Perth Children’s Hospital, Perth, WA, Australia; ^7^ Children’s Diabetes Centre, Telethon Kids Institute, The University of Western Australia, Perth, WA, Australia; ^8^ Division of Pediatrics within the Medical School, The University of Western Australia, Perth, WA, Australia; ^9^ Department of Medicine, University of Otago, Christchurch, New Zealand; ^10^ Department of Diabetes and Endocrinology, Guy’s & St Thomas’ National Health Service (NHS) Foundation Trust, London, United Kingdom; ^11^ Department of Diabetes, School of Cardiovascular, Metabolic Medicine and Sciences, King's College London, London, United Kingdom; ^12^ Institute of Diabetes, Endocrinology and Obesity, King’s Health Partners, London, United Kingdom; ^13^ Division of Endocrinology, Department of Medicine, Stanford University, Stanford, United States; ^14^ Pediatric Endocrinology and Diabetology, University Children’s Hospital, Basel, Switzerland; ^15^ Department of Clinical Research, University of Basel, Basel, Switzerland

**Keywords:** equity, type 1 diabetes, type 2 diabetes, diabetes technology, conference

## Abstract

While advancements in the treatment of diabetes continue to rapidly evolve, many of the newer technologies have financial barriers to care, opposing the egalitarian ethos of Banting who sold his patent on insulin for a nominal cost to allow it to be made widely available. Inequity in access to new therapies drives disparity in diabetes burden with potential for these gaps to widen in the future. The 2023 International Conference on Advanced Technologies and Treatments of Diabetes (ATTD) presented ground-breaking and current research in diabetes technology. Oral presentations of the ATTD conference 2023 were analyzed to describe what percentage of speakers discussed equity in their talks. Overall, less than a quarter of presenters discussed equity, though there was regional variation. To ensure that diabetes technologies reduce disparity and improve outcomes, we encourage future speakers at diabetes (technology) conferences to consider equity of diabetes care and incorporate this into their presentations.

## 1 Introduction

The 100-year anniversary of the discovery of insulin is a timely reminder that altruism is central to the ethos of diabetes management. Indeed, Banting felt it was unethical for a doctor to profit from a discovery that would save lives, leading to the insulin patent being sold to the University of Toronto for a mere $1 (1). However, in recent times, with lifesaving and burden reducing technological innovations for the management of diabetes, it is a sad indictment that profiteering from health has ensued, with a resultant health equity gap that is both intra and inter geographical (2–5).

Diabetes conferences where expert clinicians, scientists, and industry come together are a great opportunity to highlight equity, or lack of, for people living with diabetes and encourage robust discussion on how this can be addressed. Access to new diabetes technologies has been shown to improve outcomes and yet enormous disparity exists within many countries and between countries (3, 4). The International Conference on Advanced Technologies and Treatments of Diabetes (ATTD) is an annual conference where ground-breaking research in diabetes technology is shared and discussed. As one of the premier technology advancing conferences, ATTD is an excellent setting to assess how consistently equity is discussed. Hence, the aim of this study was to assess how many presentations discuss equity in diabetes technology and therapeutics.

## 2 Methods

All presentations (plenaries, parallel sessions, oral presentations, industry symposia presentations) at ATTD 2023 were assessed, either on the virtual platform, or in person by the reviewers. E-Posters were excluded.

The group of reviewers comprised nine people, five females and four males. Country of residence of most of the reviewers was in Oceania (n=8). All reviewers were given a link to a shared document containing the sessions, presentation and speaker names and country of origin of the speakers. They were asked to confirm the country of origin of the speaker, to note gender and whether the talk discussed equity or not (yes/no). They were asked to record in real-time while listening to the presentations. Equity was defined as mentioning themes around socioeconomic, access, gender and health system disparities in diabetes technology.

While it was not possible to check ethnicity of presenters, their countries as recorded from their affiliations were grouped into the following regions: North America, Western Europe, Eastern Europe, Asia, Africa, Middle East and Oceania. Session introductions by moderators were excluded from the analysis. If a single presenter gave multiple different talks, each talk was individually included in analysis. Simple descriptive statistics such as frequency and percentages were used to present data.

## 3 Results

A total of 229 speakers were included in this analysis, who contributed to a total of 266 presentations: 23 plenary presentations, 109 parallel session presentations, 103 oral presentations and 31 industry symposia presentations. For each presentation, the demographics of the speaker are presented in **Table 1**. Almost two thirds (64%) of the presentations had a male presenter, and the vast majority of speakers (86.8%) were from institutions in North America (40.6%), and Europe (46.2%).

**Table 1:** Demographics of speakers for each presentation and whether they discussed equity in their talks at the 2023 International Conference on Advanced Technologies and Treatments of Diabetes (ATTD).

	Number N (%)	Equity Discussed N/total N (%)
Overall		
	266 (100%)	63/266 (23.7%)
Gender		
Male	171 (64.3%)	43/171 (25.1%)
Female	95 (35.7%)	20/95 (21.1%)
Region		
North America	108 (40.6%)	31/108 (28.7%)
Western Europe	104 (39.1%)	18/104 (17.3%)
Eastern Europe	19 (7.1%)	2/19 (10.5%)
Oceania	4 (1.5%)	3/4 (75%)
Asia	11 (4.1%)	7/11 (63.6%)
Middle East	19 (7.1%)	1/19 (5.3%)
Africa	1 (0.4%)	1 (100%)

**Table 1** also shows the percentage of presenters that discussed equity. Overall, just under a quarter (23.7%) of presentations discussed equity in some form throughout their talk. Equity was considered by a greater percentage of presenters from institutions in Asia (63.6%), Oceania (75%) and Africa (100%), however individuals from these areas only made up 6% of presenters at ATTD 2023.

Split by presentation type, 43.5% (10/23) speakers of plenary session presentations and 33% (36/109) speakers of parallel session presentations discussed equity in their talks. In contrast only 12.6% (13/103) of the speakers of oral presentations and 12.9% (4/31) of the speakers of industry session presentations spoke of equity in their presentation.

## 4 Discussion

Fewer than a quarter of all presentations at ATTD 2023 discussed equity. Of note, presenters from institutions outside of Western Europe and North America seemed more likely to mention equity. Furthermore, speakers of plenary and parallel sessions were more likely to mention equity in their talks. However formal statistical comparisons were not performed. One has to acknowledge that presenters of short oral presentations - in contrast to presenters in industry sessions - have limited time to present their data and often need to focus of a few aspects of their research.

A further limitation of the data presented in this perspective may be that what is presented reflects primarily what the conference planning committee accepted for presentation and is not a comprehensive representation of the work being done around diabetes technology and health equity. However, new technologies have shown the greatest benefit in those from the lowest socio-economic backgrounds, and therefore have the potential to reduce disparities if equitable access is promoted (4, 6, 7). It is therefore important that all new frontiers with respect to managing diabetes strongly consider equity in their mandates. In addition, it is important to promote equitable access to diabetes therapeutics and technology by funding research through public as well as industry sectors, given that technology and insulin manufacturing companies have high annual net incomes (8–13).

If certain subsets of the population continue to miss out on new therapeutics due to financial, socio-economic or health system barriers, not only will disparities increase, but the burden of disease and subsequent complications will lead to preventable costs and strain on stressed healthcare systems worldwide (14). It is in the best interest of individuals living with diabetes, healthcare systems, and populations as a whole to reduce inequity, and the first step is to consider this in new developments. Thus, future speakers and supporters of diabetes conferences such as ATTD should be supported to consider the precedent that Banting set 100 years ago, recalling that everyone with diabetes should benefit from new innovations.

## Data availability statement

The raw data supporting the conclusions of this article will be made available by the authors, without undue reservation.

## Ethics statement

Ethical approval was not required for the study involving humans in accordance with the local legislation and institutional requirements. Written informed consent to participate in this study was not required from the participants or the participants’ legal guardians/next of kin in accordance with the national legislation and the institutional requirements.

## Author contributions

CL: Data curation, Formal Analysis, Investigation, Visualization, Writing – original draft. AA: Conceptualization, Methodology, Supervision, Writing – review & editing. JB: Investigation, Writing – review & editing. HC: Investigation, Writing – review & editing. ED: Investigation, Writing – review & editing. NH: Investigation, Writing – review & editing. SH: Investigation, Writing – review & editing. RL: Investigation, Writing – review & editing. KL: Investigation, Writing – review & editing. TW: Investigation, Writing – review & editing. MD: Conceptualization, Data curation, Formal Analysis, Investigation, Methodology, Supervision, Writing – original draft, Writing – review & editing. M-AB: Conceptualization, Data curation, Formal Analysis, Investigation, Supervision, Visualization, Writing – review & editing, Methodology.
